# *Salmonella enterica* Serovar Typhimurium Exploits Inflammation to Modify Swine Intestinal Microbiota

**DOI:** 10.3389/fcimb.2015.00106

**Published:** 2016-01-22

**Authors:** Rosanna Drumo, Michele Pesciaroli, Jessica Ruggeri, Michela Tarantino, Barbara Chirullo, Claudia Pistoia, Paola Petrucci, Nicola Martinelli, Livia Moscati, Elisabetta Manuali, Silvia Pavone, Matteo Picciolini, Serena Ammendola, Gianfranco Gabai, Andrea Battistoni, Giovanni Pezzotti, Giovanni L. Alborali, Valerio Napolioni, Paolo Pasquali, Chiara F. Magistrali

**Affiliations:** ^1^Department of Veterinary Public Health and Food Safety, Istituto Superiore di SanitàRome, Italy; ^2^Department of Comparative Biomedicine and Food Science, University of PaduaPadua, Italy; ^3^VISAVET Health Surveillance Centre, Universidad Complutense MadridMadrid, Spain; ^4^Department of Veterinary Diagnostic, Istituto Zooprofilattico Sperimentale della Lombardia e dell'Emilia RomagnaBrescia, Italy; ^5^Research and Development Area, Istituto Zooprofilattico Sperimentale dell'Umbria e della MarchePerugia, Italy; ^6^Department of Experimental Medicine, University of PerugiaPerugia, Italy; ^7^Department of Biology, University of Roma Tor VergataRome, Italy

**Keywords:** *Salmonella Typhimurium*, microbiota, inflammation, immune response, pig, salmonellosis

## Abstract

*Salmonella enterica* serovar Typhimurium is an important zoonotic gastrointestinal pathogen responsible for foodborne disease worldwide. It is a successful enteric pathogen because it has developed virulence strategies allowing it to survive in a highly inflamed intestinal environment exploiting inflammation to overcome colonization resistance provided by intestinal microbiota. In this study, we used piglets featuring an intact microbiota, which naturally develop gastroenteritis, as model for salmonellosis. We compared the effects on the intestinal microbiota induced by a wild type and an attenuated *S. Typhimurium* in order to evaluate whether the modifications are correlated with the virulence of the strain. This study showed that *Salmonella* alters microbiota in a virulence-dependent manner. We found that the wild type *S. Typhimurium* induced inflammation and a reduction of specific protecting microbiota species (SCFA-producing bacteria) normally involved in providing a barrier against pathogens. Both these effects could contribute to impair colonization resistance, increasing the host susceptibility to wild type *S. Typhimurium* colonization. In contrast, the attenuated *S. Typhimurium*, which is characterized by a reduced ability to colonize the intestine, and by a very mild inflammatory response, was unable to successfully sustain competition with the microbiota.

## Introduction

Nontyphoidal salmonellae (NTS) as *Salmonella enterica* serovar Typhimurium are a leading cause of acute food-borne zoonoses worldwide being responsible for hundreds of millions of cases of gastroenteritis and bacteremia annually (Hohmann, 2001). Pigs are important reservoir of infection for humans as they are asymptomatic carriers of broad host-range serovars of *Salmonella* (Funk and Gebreyes, 2004; Pires et al., 2011). The intestine is considered to be the biological niche of *Salmonella* with the intestinal mucosa having a central role in regulating the immune response to bacteria (Hallstrom and McCormick, 2011). However, *Salmonella* has developed strategies to overcome and cope with most of the immune defenses developed by the host (Behnsen et al., 2015). Examples of the strategies used by *Salmonella* to evade mucosal innate immunity include the ability to resist to the reactive oxygen species generated during inflammation (Bogomolnaya et al., 2013), in order to produce energy by an anaerobic respiration chain which uses an electron acceptor specifically generated in the gut under oxidative stress (Winter et al., 2010) and to resist to the sequestration of essential nutrients such as iron and zinc (Raffatellu et al., 2009; Liu et al., 2012). As a matter of fact, the ability to resist to the antimicrobial host responses characterizing gut inflammation promotes the growth of *Salmonella* in the intestinal lumen over the competing microbiota. During the past few years, there has been an expanding interest concerning the role played by intestinal microbiota in the susceptibility to enteric pathogens. Microbiota contributes to the digestion of dietary substances and to the synthesis of essential food supplements such as vitamins, and to the development or maintenance of the mucosal immune system (Littman and Pamer, 2011). Moreover, it acts as a barrier against invading bacteria both physically, blocking pathogen access to the epithelial layer, and also by outcompeting for nutrients reducing the survival and invasiveness of enteric pathogens (Hallstrom and McCormick, 2011; Sassone-Corsi and Raffatellu, 2015). However, it has been known that *S. Typhimurium* requires intestinal inflammation to circumvent “colonization resistance” provided by the intestinal microbiota (Santos et al., 2009). It has been shown that *Salmonella* can alter the normal composition of the gut microbiota, and this influence is associated with *Salmonella* virulence factors that induce inflammatory mucosal host responses (Barman et al., 2008). Furthermore, animals with disrupted microbiota have an increased susceptibility to infection (Barman et al., 2008; Juricova et al., 2013). Most of the studies examining salmonellosis have been carried out in murine models that naturally do not develop gastroenteritis. To resemble the disease in humans, mice can be subjected to antibiotic treatment in order to eliminate microbiota and to develop colitis (Ahmer and Gunn, 2011). Therefore, due to the lack of an intact microbiota, murine models are not suitable for the comprehension of the mechanisms used by *Salmonella* to thrive in the gastrointestinal environment (Elfenbein et al., 2013). To circumvent this limitation, we chose the pig as a model for our study. The advantage of the pig lies in the great similarity between humans and pigs in the gastrointestinal tract and in the disease caused by *Salmonella* as well as being a natural host of *Salmonella* (Zhang et al., 2013). We hypothesized that the Salmonella virulence degree is a determining factor in influencing the capability of the pathogen to overcome protective microbiota. To explore this, we compared the effects on the intestinal microbiota of *S. Typhimurium* wild type to that of an attenuated *Salmonella* strain lacking the ZnuABC transporter. Our findings provide evidence that the microbiota modifications induced by *Salmonella* are correlated with the virulence of the strain. Moreover, *Salmonella* could overcome colonization resistance through the reduction of microbiota members normally involved in the intestinal homeostasis and in the inhibition of pathogen growth.

## Materials and methods

### Salmonella spp. cultures

The wild-type strain *S. Typhimurium* ATCC 14028 (hereafter STM^wt^) and its isogenic attenuated *znuABC* mutant (hereafter STM^ΔznuABC^; Ammendola et al., 2007), were used throughout the study. Strains were grown overnight at 37°C in Brain Heart Infusion broth (Oxoid Ltd., Basingstoke, UK), harvested by centrifugation and washed twice in ice-cold phosphate buffer solution (PBS) (Sigma-Aldrich, Milan, Italy).

### Animals and samples collection

Thirty-one post weaned piglets old 28 days, from *Salmonella*-free sows (routinely monitored with microbiological and serological tests), were used in the experiment. Group A (9 piglets) received sterile sodium bicarbonate buffer and it was used as naïve control group. Groups B and C (11 piglets each) were orally infected with 20 ml of sterile 10% sodium bicarbonate buffer containing 2 × 10^9^ CFU of STM^ΔznuABC^ (Group B) or 2 × 10^9^ CFU of STM^wt^ (Group C). At 0, 1, 2, 7, and 12 days post infection (dpi), rectal temperature was recorded and serum and fecal samples were collected to evaluate TNF-α, IL1-α, haptoglobin, and CRP production and to detect fecal excretion of *Salmonella*, respectively. Four piglets of group A and 5 for groups B and C were sacrificed at 1 dpi, while 5 piglets of group A and 6 for groups B and C at 12 dpi. Portions of spleen, ileum, cecum, colon, ileocecal lymph nodes, and tonsil of the soft palate were taken for microbiological analysis, histology, and for mRNA isolation. Feces and cecal and colonic contents were collected to analyze the microbiota composition.

All the experiments were authorized by national authority and conducted according to European Directive (2010/63/EU; approval number 54/2012).

### Microbiology

Fecal shedding and organs colonization of STM^wt^ and STM^ΔznuABC^ were determined according to the ISO 6579: 2002/Amendment 1:2007 protocol. Briefly, samples were weighed and homogenized in nine parts of Buffered Peptone Water (BPW) (Oxoid Ltd., UK). This initial solution was then used to perform a decimal dilution series carried out by systematically transferring an aliquot of 0.5 ml of each successive dilution in 4.5 ml of BPW. All BPW-diluted samples were incubated at 37°C for 18 ± 3 h. 0.1 ml of cultures were subsequently seeded on Modified Semisolid Rappaport-Vassiliadis (MSRV) agar plates (Oxoid Ltd., UK) and incubated at 41.5°C for 24 h for the selective enrichment of *Salmonella*. A loopful of growth from each MSRV plate was streaked onto Xylose-Lysine-Desoxycholate Agar (Oxoid Ltd., UK) and Brilliant Green Agar (Oxoid Ltd., UK) plates and hence incubated at 37°C overnight. Suspect *Salmonella* colonies were subjected to biochemical identification by the BBL Enterotube II (BD Franklin Lakes, USA) and serological identification using *Salmonella* group-specific antisera (Remel, Lenexa, USA). This is a semi-quantitative approach that allows the quantification of *Salmonella* in a sample within a tenfold band (detection limit 1 CFU/g feces).

### Histology

Tissue samples of cecum were fixed in formalin, embedded in paraffin wax and stained with hematoxylin, and eosin according to standard procedures.

### Immune mediators production

TNF-α, IL1-α, haptoglobin, and C-reactive Protein (CRP) production was measured in serum samples from animals bled at 1 and 12 dpi using a sandwich ELISA (Porcine Quantikine ELISA Kit, R&D System, Mn, USA), according to the producer's instructions.

### Gene expression

Total RNA was extracted from sections of the cecum, colon, and ileocecal lymph nodes using the PureLink RNA Mini Kit (Ambion, Life Technologies). Reverse transcription of 1 μg of RNA was performed for each individual sample using Tetro cDNA Synthesis Kit (Bioline) and 5 μl of cDNA were used for real-time reaction using SensiMix II Probe Kit (Bioline). Primers for cytokines (RPL-32, IL-1α, IL-1β, TNF-α, and IFN-γ) were designed using PrimerQuest Design Tool (Integrated DNA Technologies, IDT; see Supplementary Table 1). Fold changes in gene expression were calculated using the ΔΔCt method in comparison to the results for the reference housekeeping gene RPL32.

### Fecal 16S rDNA metagenomics next-generation sequencing

Bacterial genomic DNA (gDNA) was extracted from feces, cecal, and colonic contents using QIAmp DNA Stool Mini Kit (Qiagen, Hilden, Germany). Fifty nanograms of gDNA were used to amplify by PCR the hypervariable V3-V4 regions of the 16S rDNA using bacteria/archeal primers 515F/806R with Illumina overhang adapters (Caporaso et al., 2012). One nanogram of PCR amplicon was used for each sample to prepare the sequencing library according to the Illumina Nextera XT DNA Sample Preparation Kit. During this procedure, using a limited cycle PCR, Illumina sequencing adapters, and dual-index barcodes were added to the amplicon. All the libraries were subsequently normalized and pooled by 24 prior to sequencing according to manufacturer's instructions (Illumina Nextera XT DNA Library Preparation Guide). Each pool of 24 samples was sequenced on Illumina MiSeq using a 2 × 250 paired-end (PE) setting on a standard MiSeq flow cell. Sequencing reads were trimmed and all the reads with a quality score below the Q20 parameters were discarded from the analysis. Then, all the PE reads were joined using the *join_paired_ends scripts* of QIIME utilities (Caporaso et al., 2010) to create longer fragments. The Lederhosen pipeline (based on UCLUST software and green genes v 13.5 16S database) was used to create the OTU table for each sample. The OTU tables were provided as input for the MatR package to remove singletons and to normalize the data by sequencing depth. Alpha- and beta- diversity were determined by QIIME using Shannon's and Fisher's indices for alpha diversity, unweighted Unifrac and Bray-Curtis for beta diversity, respectively.

### Quantitative real-time PCR of 16S rRNA gene sequences

q-PCR was performed using bacterial groups-specific 16S rRNA primers (see Supplementary Table 2) to determine the amount of bacteria in the study groups. However, this method is an approximation of microbial abundance as a great amount of bacteria features many copies of the 16S gene. Therefore, both variation in the abundance of organisms and genomic copy number variation can influence the quantitative prediction of 16S gene abundances. Real time PCRs were carried out on SensiMix SYBR Low-ROX Kit (Bioline). The amplification program started with an initial step at 95°C for 10 min, followed by 40 cycles of 15 s each at 95°C, 15 s at 55°C-63°C (depends on the Tm of primers), and 15s at 72°C. The 16S gene copy numbers per μl of DNA, from each sample, were determined by using standard curves generated from fragments of 16S rRNA genes of reference bacteria specific for each group cloned into plasmid (Promega) as templates. The plasmid was purified by using the Wizard Plus SV Minipreps DNA purification kit (Promega) and its concentration was quantified by using a NanoDrop® ND-1000 Spectrophotometer. With the molecular weight data of the plasmid and insert sequences, the copy number (g/molecule) was calculated according to the equation defined by Whelan et al. (2003). For each microbial population, the corresponding plasmid was chosen to create a 10-fold standard curve ranged from 10^8^ to 10^2^ copies. Copy numbers of 16S rRNA genes per μl of sample (feces, caecal, and colonic contents) were transformed into logarithms to achieve normal distribution, and the mean ± standard deviation was calculated. To estimate the copy number of *Enterobacteriaceae* other than *Salmonella*, for each sample the *Salmonella* 16S gene copy number was subtracted from the total *Enterobacteriaceae* 16S gene copy number.

### Statistical analysis

Statistical analysis was performed using GraphPad 6.0 software for Windows (GraphPad Software Inc.; San Diego; CA). Microbiota analysis by q-PCR were estimated using one-way analysis of variance (One-way ANOVA). Fecal shedding, organs colonization, and cytokines expression were analyzed using non-parametric Mann–Whitney test. Differences in body temperature and differences between groups in the TNF-α, IL1-α, haptoglobin, and CRP production were estimated using non-parametric Dunn's test. Moreover, non-parametric Kruskal–Wallis was used to test the presence of significant differences among the sample groups analyzed for each different taxonomical level considered (Phylum, Family, Genus) and Benjamini-Hochberg FDR was applied to correct multiple testing. A *P* ≤ 0.05 was considered statistically significant. Non-parametric Dunn's test was also used to estimate differences in alfa diversity.

## Results

### Pathogenicity of *Salmonella Typhimurium* is positively correlated to bacterial virulence

**Figure 1:**
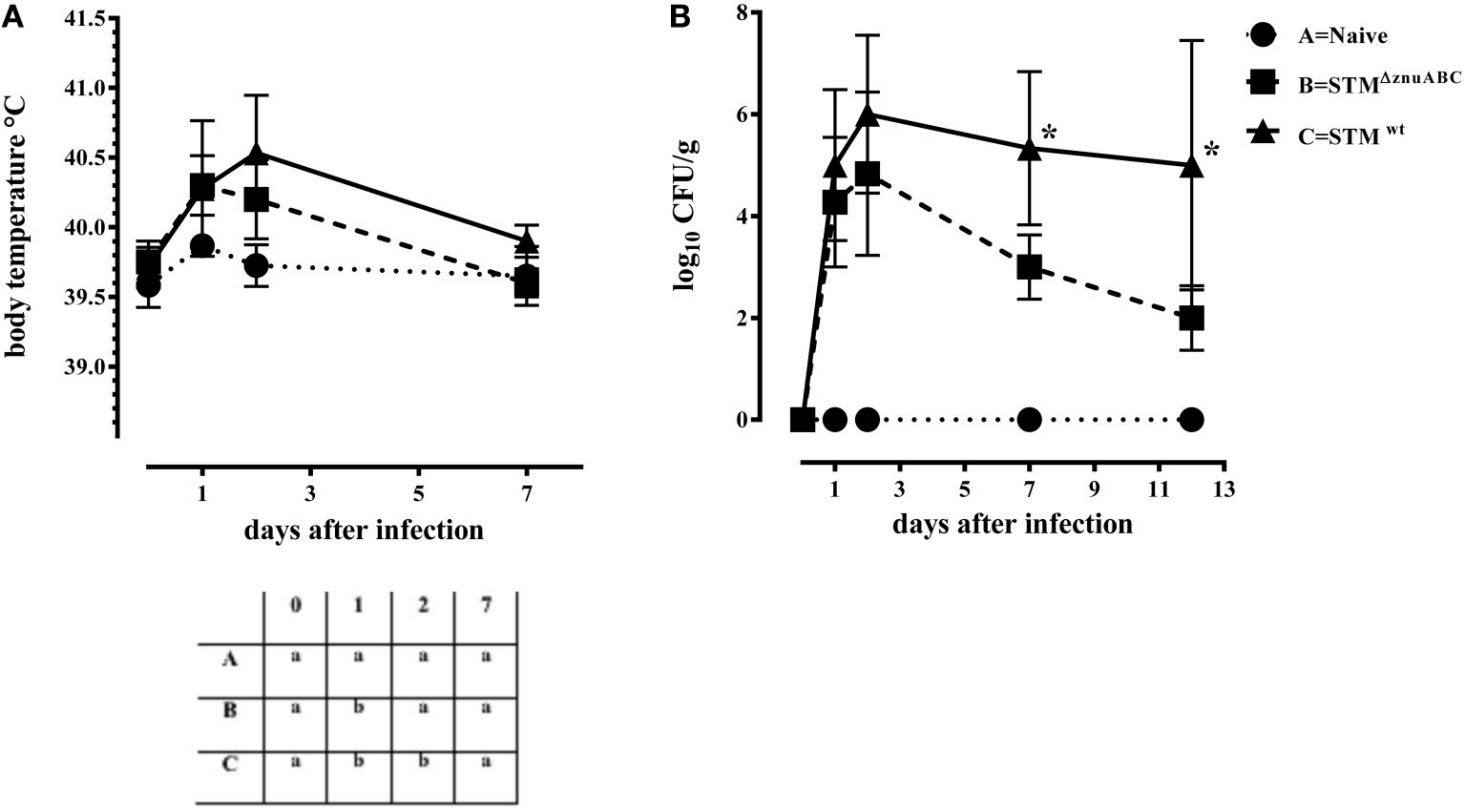
**STM^ΔznuABC^ (group B) shows a lower virulence in piglets compared to the STM^wt^ (group C)**. **(A)** Mean values and standard deviation (SD) bars of body temperature of study groups in different time points. In the table on the bottom the levels of significance were reported among groups at different time points. Different letters at each time point represent significant different results (*P* ≤ 0.05, Dunn's test). **(B)** Mean values and SD bars of CFU/g of STM^ΔznuABC^ and STM^wt^ shed in feces. Results of piglets infected with STM^ΔznuABC^ were compared to results of STM^wt^ and differences were statistically significant when ^*^P ≤ 0.05, Mann–Whitney test.

**Figure 2:**
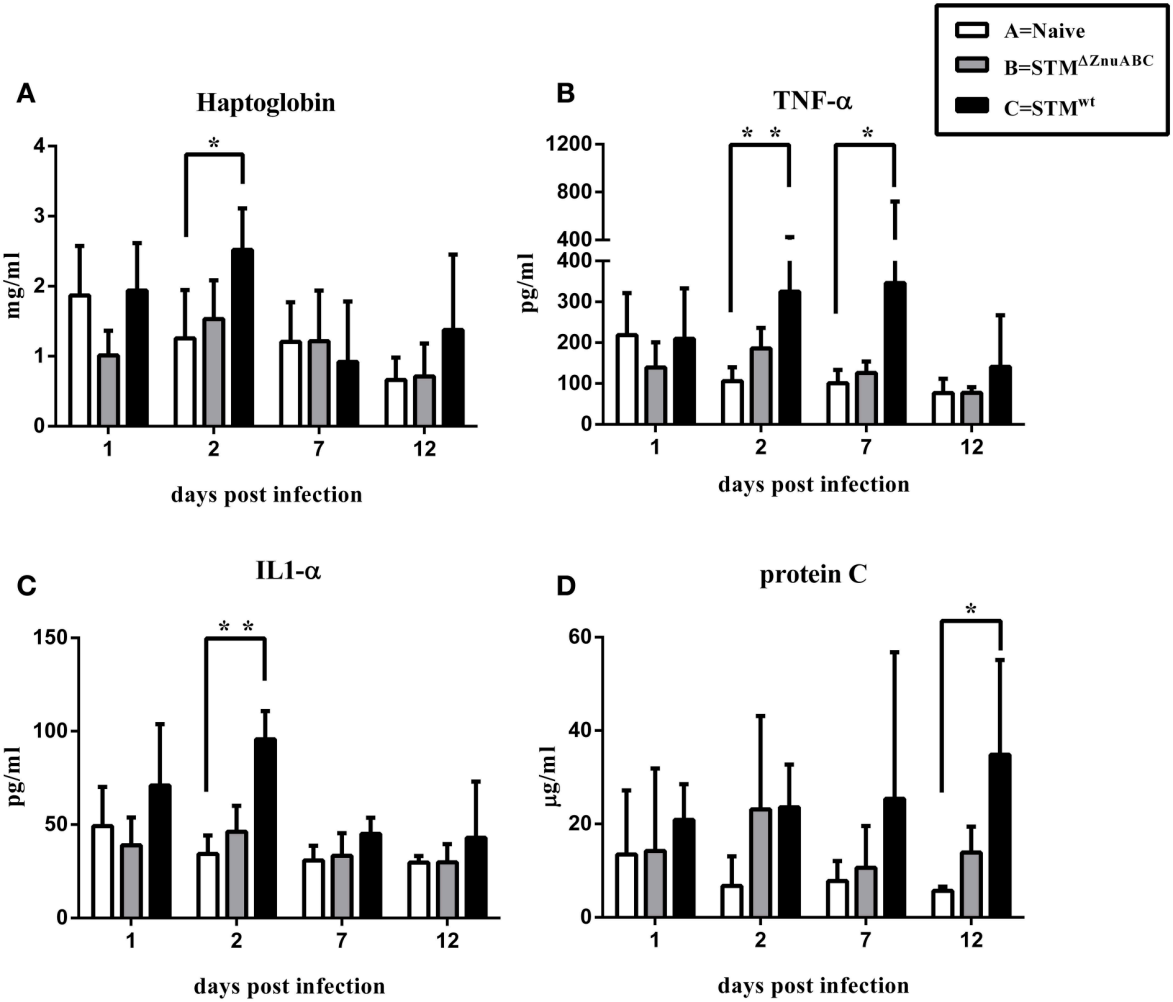
**(A–D)** *S. Typhimurium* induces an inflammatory response correlated to the virulence of the bacterial strain. Haptoglobin, TNF-α, IL1-α, and C-reactive protein levels in serum of animals were determined by ELISA. The asterisks indicate statistical significance: ^*^P ≤ 0.05 and ^**^P ≤ 0.01 (multiple comparisons-Dunn's test).

**Figure 3:**
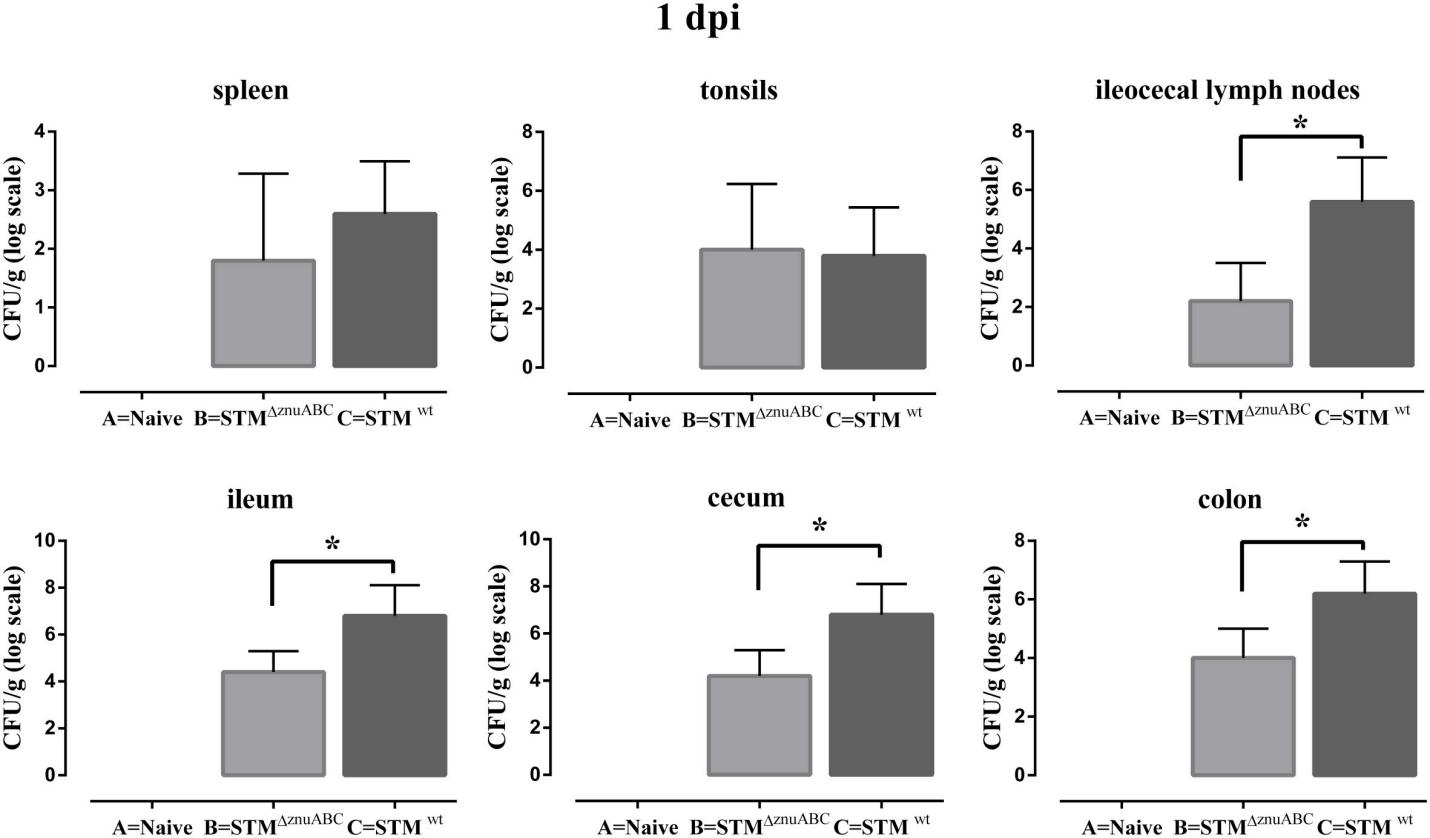
**STM^wt^ induces a higher organs colonization than STM^Δ*znuABC*^ at 1 dpi**. Piglets were orally infected with 2 × 10^9^ CFU of STM^ΔznuABC^ (group B) or STM^wt^ (group C), and bacterial burdens were determined at 1 dpi. Differences between groups B and C were estimated using non-parametric Mann–Whitney test and were considered significant when ^*^P ≤ 0.05. Organ samples taken from naïve animals (group A) were negative. Error bars represent one SD from the mean.

Piglets infected with STM^ΔznuABC^ (group B) and STM^wt^ (group C) had a transient increase in body temperature at 1 dpi compared with naïve controls (group A). At 2 dpi, only the group C (STM^wt^) continued showing a significantly higher body temperature than group A (Figure 1A). Moreover, differences in the levels of *Salmonella* fecal shedding were observed among the study groups. Animals infected with STM^wt^ and STM^ΔznuABC^ started to shed bacteria the day after experimental infection and reached the peak of excretion at 2 dpi. However, unlike group C (STM^wt^) that continued shedding a similar amount of bacteria throughout the whole period of observation, group B (STM^ΔznuABC^) showed a sharp decline over time (Figure 1B). To further assess the inflammatory response induced by STM^wt^ and STM^ΔznuABC^, piglets were bled at different time points and haptoglobin, CRP, IL1-α, and TNF-α levels were measured in sera. Group C (STM^wt^) had an early immune response characterized by a significant increase of haptoglobin and IL1-α at 2 dpi, and TNF-α at 2 and 7 dpi, followed by a late production of CRP which reached a significant level at 12 dpi. Conversely, group B (STM^ΔznuABC^) did not show any different production of haptoglobin, CRP, IL1-α, and TNF-α when compared with the naïve (group A; Figure 2). Piglets of different groups were euthanized at 1 and 12 dpi to assess bacterial colonization of organs. As shown in Figure 3, colonization occurred as early as 1 dpi, either in gut or in systemic organs. However, piglets infected with STM^wt^ (group C) showed a significant higher degree of colonization than piglets infected with STM^ΔznuABC^ (group B) in the gut organs (*p* < 0.05) at 1 dpi (Figure 3) and in the colon (*p* < 0.05) at 12 dpi (Supplementary Figure 1). Organs samples taken from naïve animals (group A) were negative.

These findings confirm that STM^wt^ and STM^ΔznuABC^ have a differential colonization efficiency. Moreover, STM^ΔznuABC^ did not show a significant systemic inflammation. We could infer that these results are a direct consequence of the intrinsic incapability of STM^ΔznuABC^ to induce an inflammatory response but, in alternative, they could be due to the lower colonization of STM^ΔznuABC^ which is not sufficient to give rise to a systemic immune response.

### Histology

**Figure 4:**
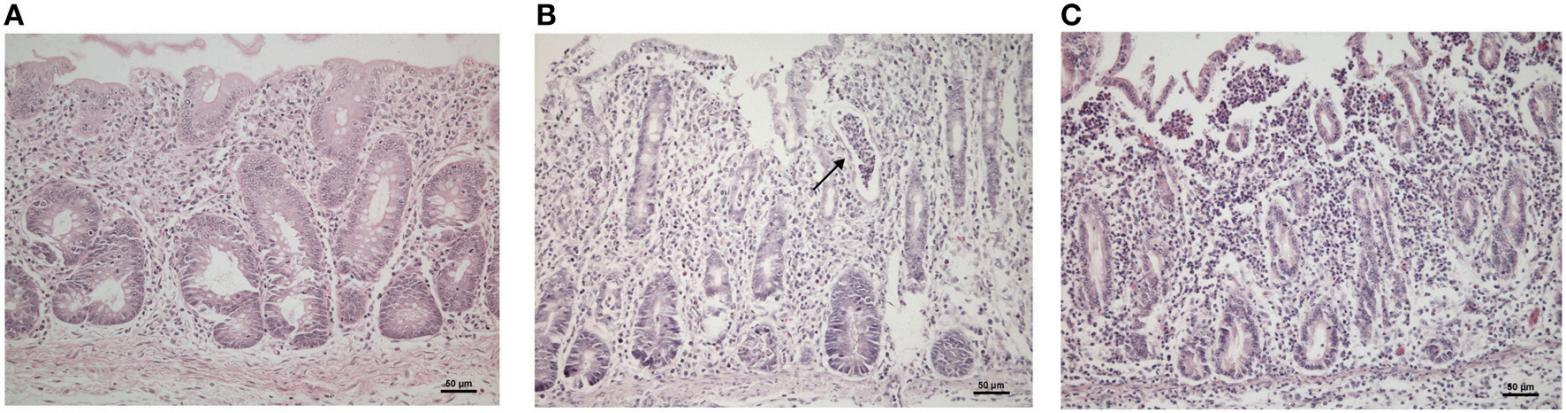
**(A–C)** Photomicrographies showing histological changes of the cecum. **(A)** Naïve control piglets; **(B)** piglets infected with STM ^ΔznuABC^: multifocal and moderate neutrophilic infiltrate (arrows), crypt abscess formation; **(C)** piglets infected with STM^wt^: marked and diffuse neutrophilic infiltration.

We compared the cecum histopathological findings from control, STM^ΔznuABC^ and STM^wt^-infected piglets at 1 and 12 dpi. At 1 dpi, sections from control piglets did not show inflammatory infiltrate (Figure 4A); conversely, piglets infected with STM^ΔznuABC^ and STM^wt^ showed neutrophilic infiltrate in the lamina propria and submucosa (Figures 4B,C). The neutrophilic infiltrate appeared moderate and multifocal in the STM^ΔznuABC^ (Figure 4B), with crypt abscess formation, whereas marked and diffused in the STM^wt^ infected piglets (Figure 4C). On the other hand, the neutrophil inflammation was mild at 12 dpi and present in a multifocal pattern in piglets infected with STM^ΔznuABC^, while inflammation was mild and diffuse in piglets infected with STM^wt^ (data not shown). Overall, a histological investigation indicated the presence of inflammatory infiltrate only in STM^wt^ and STM^ΔznuABC^. A higher degree of inflammation was observed in piglets infected with STM^wt^.

### Influence of *Salmonella* infection on the expression of pro-inflammatory cytokines

**Figure 5:**
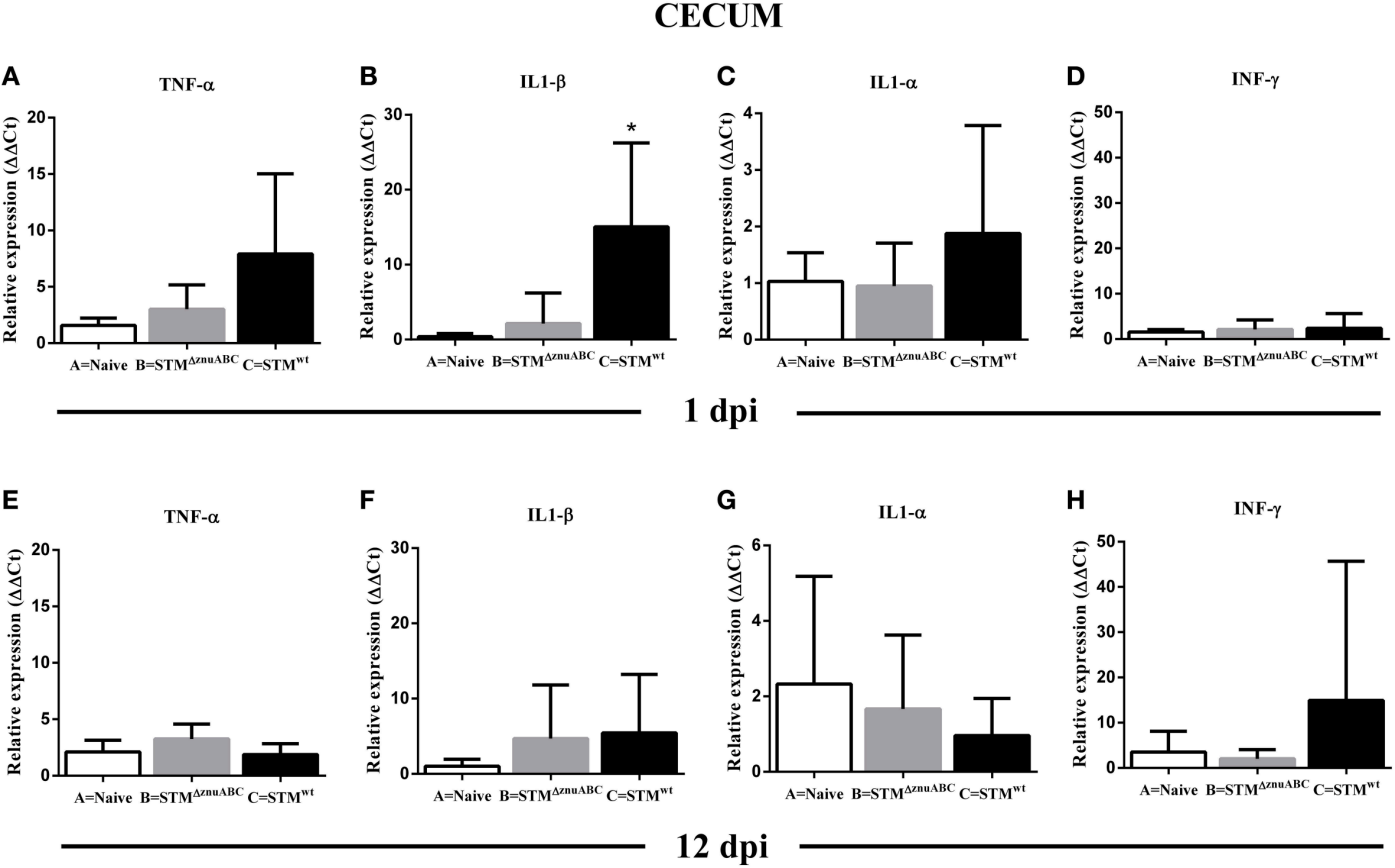
**(A–H)** Cytokines expression reveals that unlike STM^wt^, STM^ΔznuABC^ strain is not able to induce a strong host immune response. TNF-α, IL1-α, IL1-β, and INF-γ expression was measured in the cecum at 1 and 12 dpi by real time RT-PCR. Gray bars and black bars represent STM^ΔznuABC^-infected (group B) and STM^wt^-infected piglets (group C), respectively. The asterisk indicates statistical significance ^*^P ≤ 0.05, Mann–Whitney test.

Pro-inflammatory (IL1-α, IL1-β, TNF-α) and regulatory (IFN-γ) cytokines were observed so as to evaluate the early immune response in the ileocecal lymph nodes, colon, and cecum at 1 and 12 dpi (Figures 5A–H; Supplementary Figures 2A–H, 3A,B). At 1 dpi, we observed a tendency of the pro-inflammatory cytokines to increase in all organs analyzed; however, only the increase of IL1-β (*p* < 0.05) in the cecum and in the colon, and IL1-α (*p* < 0.05) in the lymph nodes of group C (STM^wt^) were statistically significant (Figures 5A–C; Supplementary Figures 2A–C, 3A–C). At 12 dpi, overall expression of IL1-α, IL1-β, and TNF-α returned to baseline levels (Figures 5E–G; Supplementary Figures 2E–G, 3E–G). Moreover, TNF-α (*p* < 0.01), IL1-β (*p* < 0.01), and IL1-α (*p* < 0.05) were significantly down-regulated in the colon of piglets infected with STM^ΔznuABC^ (group B; Supplementary Figures 2E–G), and IL1-α (*p* < 0.05) also in the lymph nodes of group C (STM^wt^; Supplementary Figures 3E–G).

### *S. Typhimurium* alters composition of the microbiota in the post-weaned piglets model

**Figure 6:**
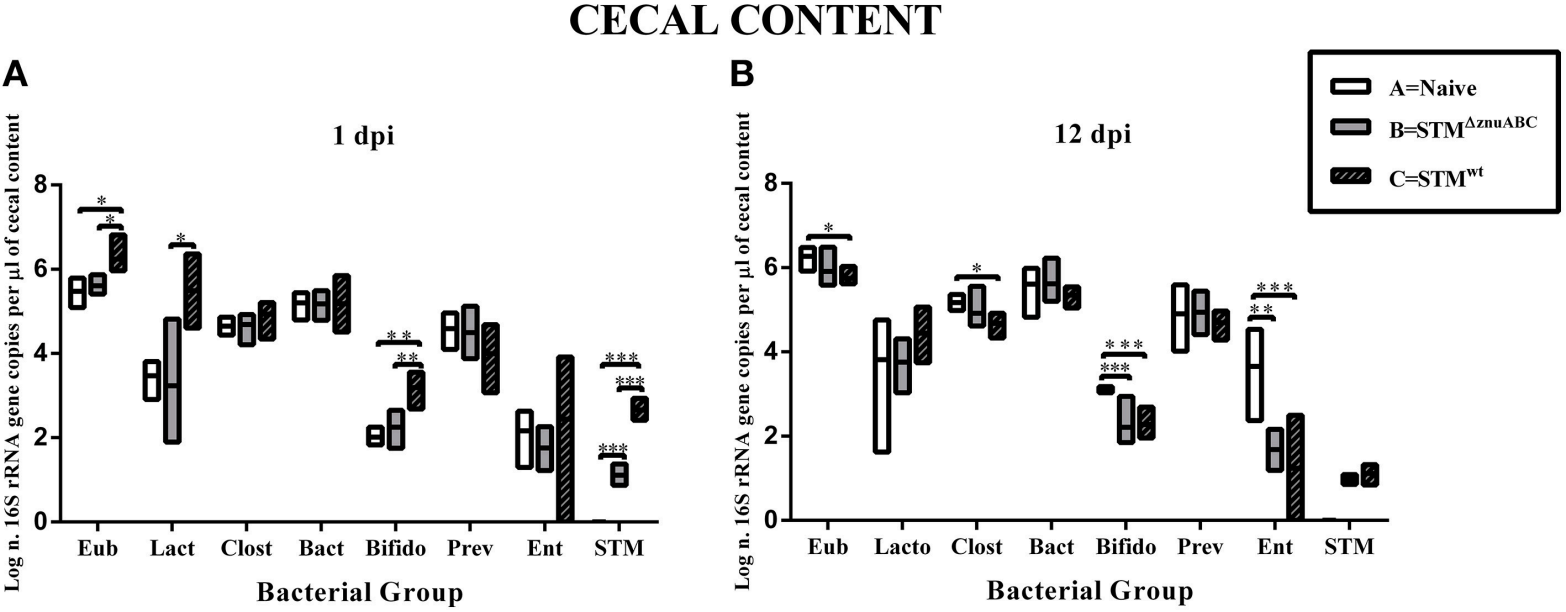
**STM^wt^ and STM^ΔznuABC^ differently modify cecal microbiota of piglets**. Piglets were sacrificed at 1 and 12 dpi **(A,B)**. Bacterial genomic DNA was isolated from cecal content and qPCR analysis measured the abundance of specific commensal bacterial groups. White bars represent uninfected controls (group A). Gray and gray-black bars represent STM^ΔznuABC^- (group B) and STM^wt^- (group C) infected piglets, respectively. *P*-values were calculated using One-way ANOVA with Bonferroni's post-test. Significant differences between groups are indicated by ^*^P ≤ 0.05, ^**^P ≤ 0.01, and ^***^P ≤ 0.001. Eub, all bacteria; Lacto, *Lactobacillus/Lactococcus* group; Clost, *Eubacterium rectale/Clostridium coccoides*; Bact, *Bacteroides* sp.; Bifido, *Bifidobacterium*; Prev, *Prevotellaceae*; Ent, *Enterobacteriaceae other than Salmonella; STM, S. Typhimurium.*

Aiming to more specifically analyze the impact of STM^wt^ and STM^ΔznuABC^ on some of the most representative bacterial members, we used quantitative real time PCR (q-PCR). As depicted in Figure 6, consistent changes in the microbiota were present primarily in the cecal contents at 1 day post-*Salmonella* infection, with a significant increase of total 16S rRNA gene copies (representative of total bacterial numbers) in piglets infected with STM^wt^ (group C; *p* < 0.05) compared to naïve animals (group A) and piglets infected with STM^ΔznuABC^ (group B; *p* < 0.05). Differences in the *Lactobacillus/Lactococcus* group were statistically significant between groups B and C (*p* < 0.05) and very close to significance between groups A (naïve) and C (STM^wt^) in the cecum (Figure 6A). In the feces (Supplementary Figure 4), the *Lactobacillus/Lactococcus* group showed significant differences at 1, 2, 7, and 12 dpi (*p* < 0.05) between groups A and C, and only at 12 dpi between groups B and C (*p* < 0.05; Supplementary Figure 4). A decrease in the *Eubacterium rectale*/*Clostridium coccoides* group was evident in group C (*p* < 0.05) at 12 dpi in the cecum and at 2 dpi in the feces (*p* < 0.01; Figure 6B; Supplementary Figure 4C). No differences among the three experimental groups were observed for *Bacteroides* in any of the samples analyzed. Conversely, at 1 dpi an evident increase in the *Bifidobacterium* group was observed in all the districts investigated between groups A and C (*p* < 0.01 for cecal content and *p* < 0.001 for colon and feces) and between groups B and C (*p* < 0.01; Figure 6A; Supplementary Figures 4B, 5A). At 12 dpi, the *Bifidobacterium* group showed a sharp reduction in groups B (*p* < 0.001) and C (*p* < 0.001) in the cecal content and in group B (*p* < 0.05) in the colonic one when compared to group A (naïve; Figure 6B; Supplementary Figure 5B). The levels of the *Enterobacteriaceae* other than *Salmonella* decreased significantly in both groups of animals infected with *Salmonella* strains in the cecal and colonic contents at 12 dpi (Figure 6B; Supplementary Figure 5B). A higher level of *Salmonella*, consistent with the microbiological findings, was observed in group C (STM^wt^) compared to group B (STM^ΔznuABC^) in all the intestinal samples, while *Salmonella* was never detected in group A (naïve; Figure 6; Supplementary Figures 4, 5).

These results show that *S*. Thyphimurium is able to alter intestinal microbiota in pigs inducing modifications correlated to its virulence.

### Bacterial diversity of the fecal microbiota after *Salmonella* infection

Massive parallel sequencing of the 16S rDNA hypervariable V3-V4 region was performed on fecal samples available from the three experimental groups A, B, and C. The sequencing yielded a total of 177198 reads passing quality control (median reads per sample 11030). OTU classification yielded a median of 5742 OTUs per sample. Sequencing reads are available at http://www.ncbi.nlm.nih.gov/bioproject/PRJNA302126 (BioProject accession ID: PRJNA302126).

**Figure 7:**
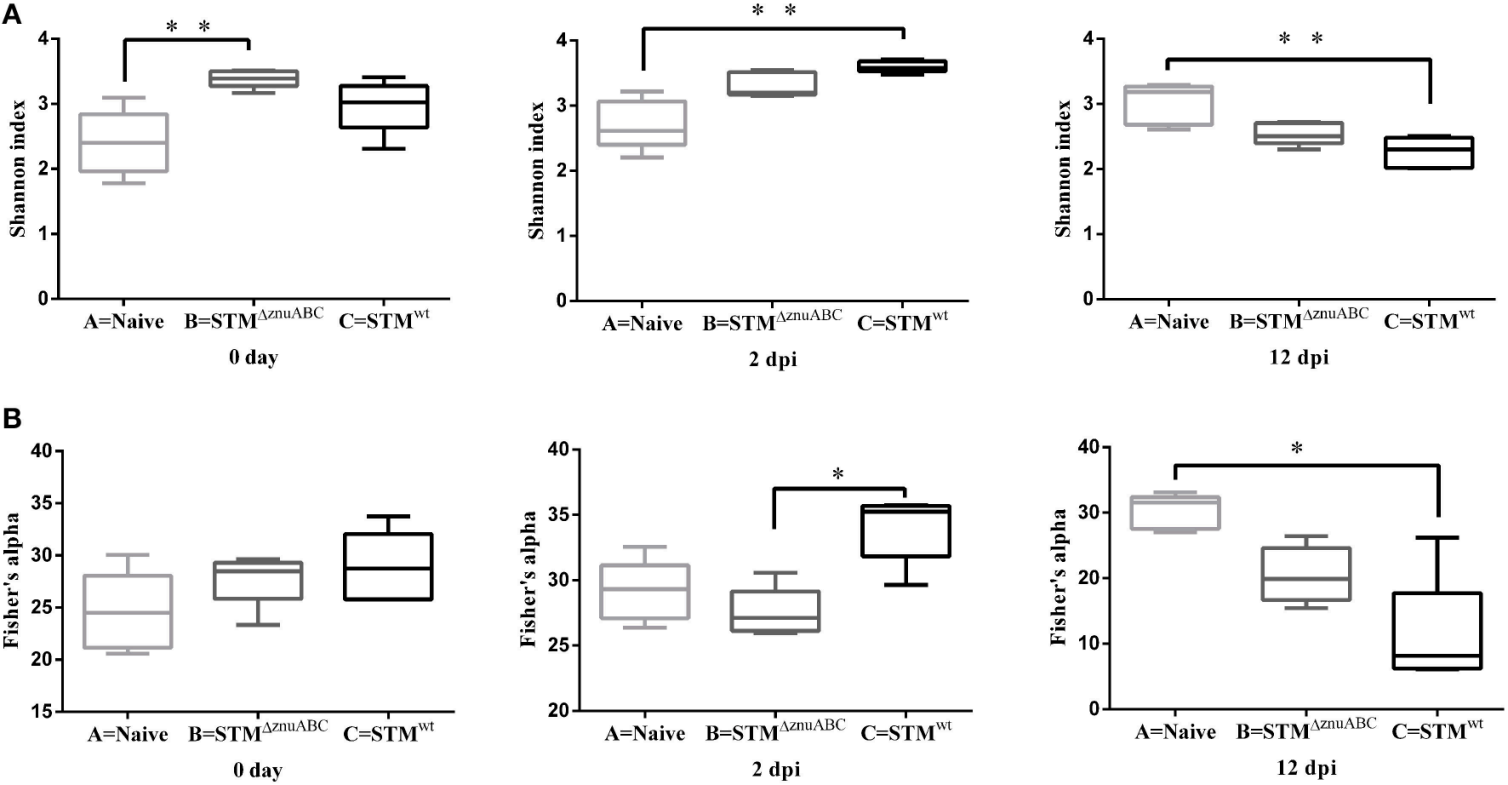
**Structural comparison of fecal microbiota among groups A, B, and C**. The Shannon index **(A)** and Fisher92s alpha **(B)** were used to estimate diversity of the fecal microbiota in naïve animals (group A) and in STM^ΔznuABC^- (group B) and STM^wt^- (group C) infected piglets. Boxes represent median, and first and third quartiles; whiskers indicate minimum and maximum values. The asterisks indicate statistical significance ^*^P ≤ 0.05 and ^**^P ≤ 0.01, Dunn's test.

We evaluated the bacterial diversity of the fecal microbiota associated with *Salmonella* strains by estimating alpha- and beta- diversity. Shannon index demonstrated that the fecal microbiota diversity in piglets infected with STM^ΔznuABC^ (group B) and STM^wt^ (group C) was significantly higher than the naïve animals (group A), at 0 and at 2 dpi respectively (*p* < 0.01). However, group C showed a significant lower alfa diversity at 12 dpi than group A (Figure 7A). Using Fisher's alpha, an index not influenced by the sample size and less affected by the abundance of the most common species than Shannon's index, we found a higher diversity in piglets belonging to group C compared to group B at 2 dpi (*p* < 0.05). At the same time, Fisher's alpha confirmed the significant lower alfa diversity in group C at 12 dpi compared to group A (*p* < 0.05; Figure 7B).

**Figure 8:**
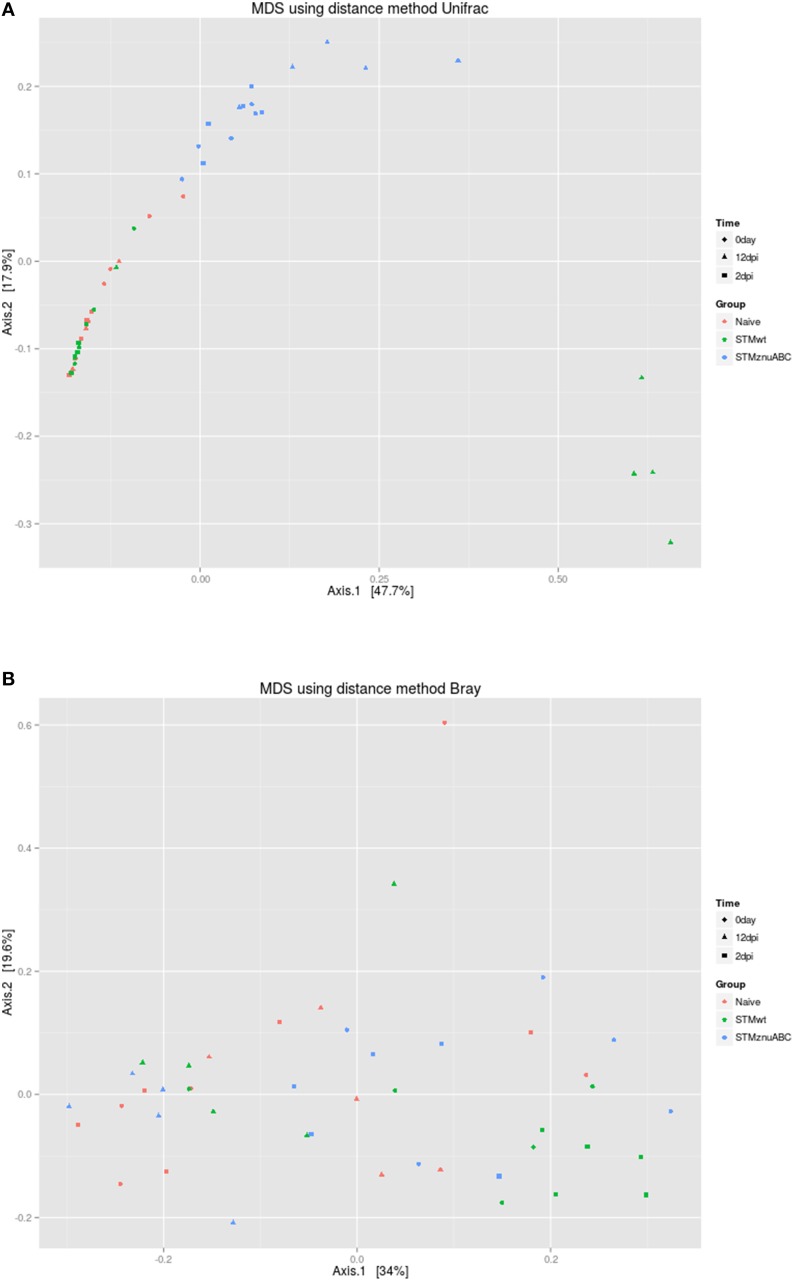
**Principal Coordinate analysis plot (PCoA) of unweighted UniFrac distances (A) and Bray-Curtis dissimilarity (B) for the fecal microbiota across the three study groups**. PCA, principal coordinate.

The beta diversity was calculated using both unweighted Unifrac and Bray-Curtis dissimilarity; principal component analysis (PCoA) was performed. As shown in Figure 8A, using Unifrac, four out of five samples belonging to group C (STM^wt^) clustered separately along the principal coordinate 1 (PCA1) at 12 dpi. In addition, a clear separation of group B (STM^ΔznuABC^) from the rest of the samples is noticeable along the principal coordinate 2 (PCA2). The PCoA using Bray-Curtis dissimilarity did not allow any clear separation of the groups, although all the five samples belonging to group C (STM^wt^), at 2 dpi, clustered at the extreme right along the principal coordinate 1 (PCA1; Figure 8B). On the light of these data, it can inferred that Shannon and Unifrac results, in which it seem to be differences among groups at time 0, could be biased by small sample size. Therefore, the microbiota composition of the different groups could be considered similar at time 0.

### *Salmonella* strains-associated alterations in fecal microbiota by NGS

**Figure 9:**
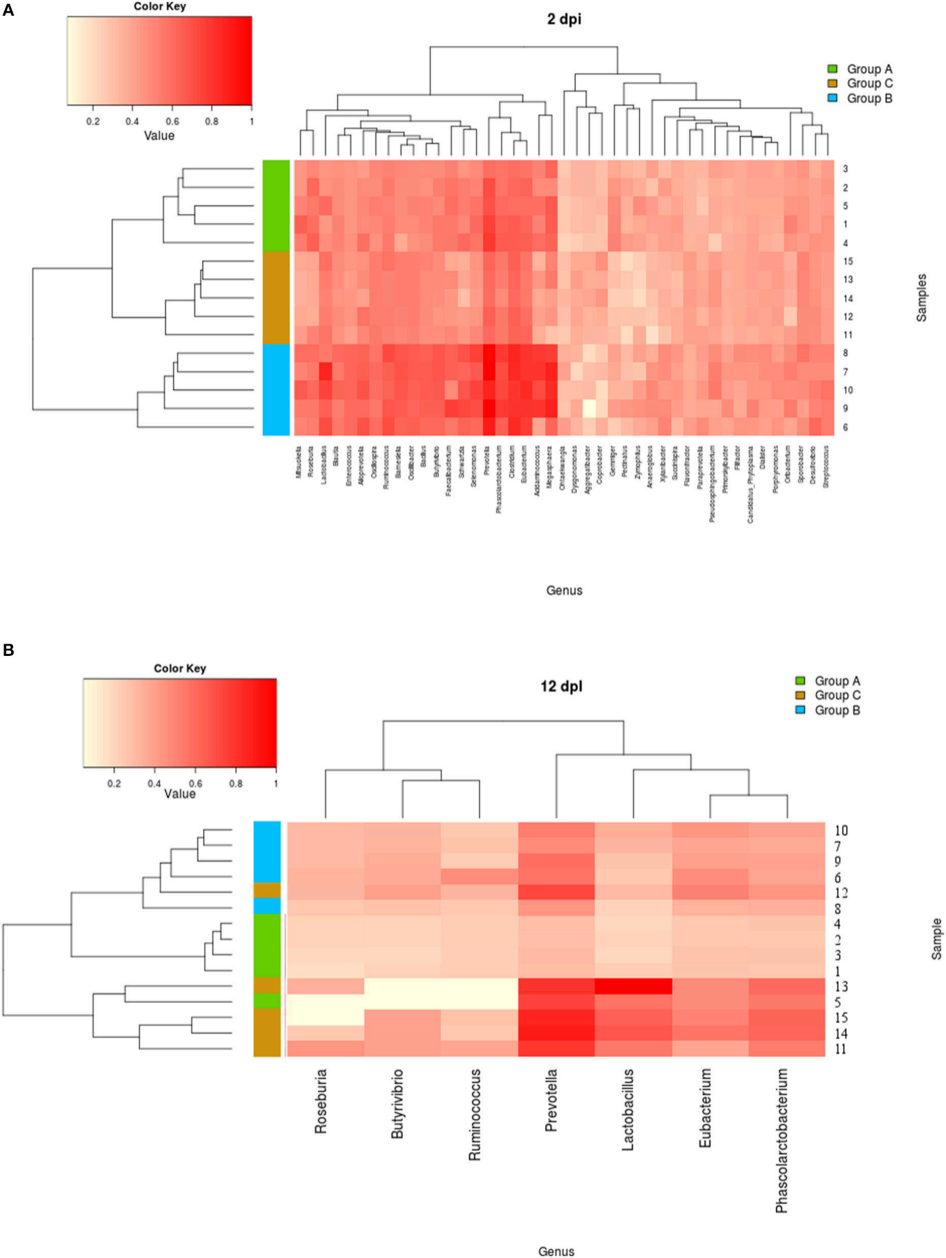
**(A,B)** Heatmap indicating genus-level changes in the microbiota composition of piglets Naïve (group A), and piglets infected with STM^ΔznuABC^ (group B) or with STM^wt^ (group C) at 2 and 12 dpi. The relative abundance of the most represented genera is indicated by a gradient of color from white (low abundance) to red (high abundance). The hierarchical clustering analysis of the samples, based on the similarity of the microbiota composition, are displayed on the left. Animals 1–5: group A (Naïve), green; animals 6–10: group B (STM^ΔznuABC^), blue; piglets 11–15: group C (STM^wt^), orange.

In order to compare how the composition of the fecal bacteria differed among treatment groups, the Kruskal–Wallis test and the Benjamini-Hochberg FDR method were used to identify differentially abundant taxa. Genus-level normalized data are available in Supplementary Table 3. The distribution of reads into phyla revealed that the bacterial communities of all samples consisted primarily of *Firmicutes* and *Bacteroidetes*. Microbiota analysis showed that 7 phyla, 112 families, 404 genera, and 15 phyla, 143 families, and 719 genera differed across groups A, B, and C, respectively at 2 and 12 dpi (see Supplementary Table 4). Figures 9A,B and Supplementary Figure 6 represent heatmaps showing the genus-level clustering according to frequency within each sample (abundance ratio greater than 0.1) at times 0, 2, and 12 dpi; abundant genera were color coded red, and white color coding indicated missing genera. The most remarkable difference reported in the piglets infected with STM^wt^ (group C) compared with naïve (group A) is that they showed an abundant presence of lactic acid-producing bacteria and a reduction of short chain fatty acids (SCFAs)-producing bacteria (Figures 9A,B). Analysis of data also revealed that piglets infected with STM^wt^ (group C) initially showed a decrease in Prevotella at 2 dpi compared to the naïve (group A). In addition, at 12 dpi, a more abundant presence of Prevotella, Phascolarcobacterium, and Eubacterium was evident in group C (STM^wt^) rather than in groups A and B.

Moreover, clustering analysis highlighted the differences in the sample distributions according to the treatment type. At 2 dpi, the most represented genera displayed a perfect clusterization of each single sample into its belonging study group (Figure 9A). Similarly, at 12 dpi, each piglet grouped into its belonging treatment group, except 2 samples (5 and 12) clustered in a different study group (Figure 9B). Moreover, at 12 dpi, groups A (naïve) and B (STM^ΔznuABC^) are more similar to each other, while group C (STM^wt^) featured more relevant effects (Figure 9B). No significant differences were detected when each single group was analyzed longitudinally according to the three collection times. These data show that infection with different strains of *S. Typhimurium* is associated with different alterations of fecal microbiota.

## Discussion

The importance of pigs as a source of *Salmonella* in the food chain is well-known. However, while *Salmonella* pathogenicity has been widely studied in mice, our knowledge on the modality of interaction of this pathogen with pigs is still limited. It has been known that different and multiple factors can influence the dynamics of *Salmonella* colonization in swine, including pathogen features (virulence mechanisms, exposure dosage), pig immune responses and the complex interplay between the pathogen and the intestinal microbiota (Bearson et al., 2013). In this study, we used a post-weaned piglet model to compare differences in the host colonization, inflammatory response, and modification of the intestinal microbiota induced by STM^wt^ and STM^ΔznuABC^ in order to elucidate the interplay among host, pathogen and gut microbiota. STM^ΔznuABC^ was chosen due to the fact that our previous studies have revealed that this strain is strongly attenuated either in mice or in pigs (Ammendola et al., 2007; Pasquali et al., 2008; Pesciaroli et al., 2013). Moreover, studies carried out in a mouse model showed that ZnuABC-mediated zinc uptake confers resistance to the antimicrobial protein calprotectin and promotes *Salmonella* growth over the competing intestinal microbiota (Liu et al., 2012).

Here, we demonstrate that a different organs colonization, intestinal inflammation and modification of porcine microbiota are correlated with the different virulence of *Salmonella* strains. The inflammatory response evaluated through the expression of the immune mediators, and corroborated by histological findings, has shown that STM^wt^ induces a prompt increase of serum markers of inflammation during the early stage of infection (1 dpi). Moreover, at the same time point, the expression of tissue-associated markers showed a tendency to increase even if only IL1-β in cecum and colon (*p* < 0.01) and IL1-α in ileocecal lymph nodes (*p* < 0.01) reach statistical significance. The prompt induction of host response could be due to the rapid and high-level replication of STM^wt^ as showed by our microbiological data. On the contrary, at 1 dpi, the histological and immunological analysis revealed a mild intestinal inflammation and a poor systemic response induced by STM^ΔznuABC^ confirming characteristics of attenuation in growth and virulence of this strain. As a whole, these observations indicate that the host is able to mount a rapid innate immune response following *Salmonella* infection within a few hours after gut colonization. The magnitude of the response and the severity of the clinical manifestations provide evidence that the host response and lesions are correlated and dependent to *S. Typhimurium* virulence.

It is known that, similarly to what happens *in vitro* and in murine models of infection (Barthel et al., 2003; Stecher et al., 2007; Barman et al., 2008), *S. Typhimurium* strains induce an acute inflammatory response in the intestinal mucosa also in piglets (Bearson et al., 2013). Several studies have proved how *S. Typhimurium* takes advantages of inflammation to compete with the resident microbiota and to colonize the inflamed gut in mice (Lupp et al., 2007; Stecher et al., 2007; Barman et al., 2008; Winter et al., 2010) and piglets (Chirullo et al., 2015). In our study, we investigated the impact of *S. Typhimurium* on the porcine intestinal microbial communities. We found that *S. Typhimurium* infection modifies either the number or the composition of gut resident bacteria. In particular, these changes were associated with STM^wt^, while the attenuated STM^ΔznuABC^ seemed to be less fit to sustain competition with the microbiota. These observations are in agreement with the studies performed in mice, where attenuated *Salmonella* mutants do not colonize intestine as well as wild-type strains as they are not able to trigger an efficient inflammatory response (Stecher et al., 2007; Lawley et al., 2008; Raffatellu et al., 2009; Winter et al., 2010).

The major changes in the microbiota composition are mainly related to the significantly more abundant presence of *Lactobacillus/Lactococcus* group after STM^wt^ infection. This observation is in agreement with the results obtained by Videnska et al. (2013), which showed a significant increase of Lactobacillaceae in chicken cecal microbiota after *S*. Enteritidis infection. A possible explanation could be attributable to the microaerophilic growth of Lactobacilli, which may allow them to survive under conditions of increased redox potential due to the production of reactive oxygen species by granulocytes infiltrating the site of inflammation as occurs in a highly inflamed gut (Videnska et al., 2013). Indeed, there is evidence that lactic acid accumulation could contribute to impair the intestinal defense barrier and increase the osmotic load in the intestinal lumen (Ling et al., 2014). The utilization of next-generation high-throughput sequencing allowed a wider description of the intestinal microbiota. In our study, clustering analysis shows that the microbiota composition changed after infection with *Salmonella* strains and the characteristics of the modifications were correlated with the virulence of the strain used. Our analysis reveals a different abundance of the most represented genera in piglets infected with STM^wt^ when compared with STM^ΔznuABC^ and naïve piglets. In fact, microbiota of piglets infected with STM^wt^ was characterized by an overall reduction of SCFA-producing bacteria (*Ruminococcaceae* including *Faecalibacterium, Roseburia, Butyrivibrio*, and *Clostridium* genera). SCFAs such as acetate and butyric acid are produced by microbial fermentation of carbohydrates and provide beneficial immunomodulatory and anti-inflammatory properties (Ling et al., 2014). In particular, butyric acid contributes to the inhibition of *Salmonella* in an acidic environment (Bearson et al., 2006), decreases invasion of intestinal cells by down-regulating expression of Pathogenicity island 1 (Gantois et al., 2006) and reduces the *Salmonella*-induced proinflammatory response of enterocytic cells *in vitro* (Malago et al., 2005). In line with these observations, previous studies showed that *Faecalibacterium*, which is correlated with butyrate production, also exhibits anti-inflammatory effects counterbalancing intestinal microbiota dysbiosis (Sokol et al., 2008). Hence, the reduced abundance of SCFA-producing bacteria induced by STM^wt^ could explain the enhanced inflammatory status observed in the gastrointestinal tract of piglets treated with this *Salmonella* strain; and it could be of interest to investigate the mechanisms leading to a reduction of this potentially protective component of the intestinal microbiota. Moreover, upon infection with *Salmonella* strains, microbiota composition also showed changes in *Prevotella, Phascolarcobacterium and Eubacterium*. Similarly to what elsewhere reported (Bearson et al., 2013), we observed a decrease of *Prevotella* in piglets infected with STM^wt^ at 2 dpi. However the limitation of available information about the biological function of such genera makes difficult to extrapolate any significant meaning to our findings. At the same time it should be acknowledged that the alpha and beta diversity patterns across the three groups within the three time points analyzed presented several discrepancies that can be attributable to the sensitivity of the next-generation sequencing technology and to the relative small sample size. However, both alpha-diversity indices converge on a significant lower alpha-diversity in group C compared to group A at dpi 12. At the same time, the significant difference found in the whole microbiome composition at time 0 between group A and group B, highlighted by Shannon alpha index and Unifrac beta-diversity PCoA, may raise the possibility that the inability of the mutant strain to colonize the intestine could be related to the composition of the microflora. Although we cannot discard this hypothesis, the present data does not allow any speculation and further studies using a larger sample size and, possibly, a more detailed time-course is warranted. Overall, our data show that the results of the interaction among *Salmonella*, the intestinal microbiota and the gut response are influenced by the specific characteristics of the three factors. The virulence of *Salmonella* and the alteration of microbiota composition is crucial in determining the outcome of the infection.

## Author contributions

RD planned and performed the research, analyzed data and wrote the manuscript. PP designed and planned the research, participated to the interpretation and discussion of the results, and revised the paper; CM planned and performed the research, analyzed data and revised the manuscript. MP, BC, and MT performed part of the research and revised the manuscript. GA, JR, and NM performed part of the research. SA and AB revised the manuscript. GG revised the manuscript. GP and LM performed part of the research and contributed to the analysis of the data. EM and SP performed histological analysis. VN and MP performed next-generation sequencing and analyzed data. PP and CP contributed to perform technical experiments.

## Funding

This work was supported by ISS intramural research funds and by Transnational Research Project EMIDA ERA-NET “HealthyGut–Multi-focal strategies to improve gut health and reduce enteritis in poultry and pigs”(MIPAF–DM 27373/7303/2010).

### Conflict of interest statement

The authors declare that the research was conducted in the absence of any commercial or financial relationships that could be construed as a potential conflict of interest.
