# Supplementary material for: Salmonella enterica Serovar Typhimurium Exploits Inflammation to Modify Swine Intestinal Microbiota
Source: Front Cell Infect Microbiol. 2016 Jan 22;5:106. doi: 10.3389/fcimb.2015.00106 (PMC4722131; doi:10.3389/fcimb.2015.00106)
Supplement: Supplementary file 1 [file Table1.docx]

**Supplementary Table 1.** Oligonucleotide sequences and DNA probes for the detection of porcine cytokines. F and R indicate primers forward and reverse.

| mRNA target | Oligonucleotides (5’-3’) |
| --- | --- |
|  | F: CCTACTGCACTTCGAGGTTATC |
| **TNF-α** | R: ACGGGCTTATCTGAGGTTTG |
|  | Probe: 56-FAM/CTGGCCCAA/ZEN/GGACTCAGATCATCG/IBFQ |
|  | F: AGACCAGTCTCCTCTTCTTCT |
| **IL1-α** | R: CCTGCCTTGTGGCAATAAAC |
|  | Probe: 56-FAM/TACTTCAAA/ZEN/TCAGCCGCCCATCCA/IBFQ |
|  | F: GACCTTAGGGATCAAGGGAAAG |
| **IL1-β** | R: CCATGTCCCTCTTTGGGTATC |
|  | Probe: 56-FAM/TGATGAAAG/ZEN/ATAACACGCCCACCCT/IBFQ |
|  | F: TCAAAGATAACCAGGCCATTCA |
| **IFN-γ** | R: CAGTTTCCCAGAGCTACCATTTA |
|  | Probe: 56-FAM/AGGAGCATG/ZEN/GATGTGATCAAGCAAGA/IBFQ |
|  | F: CTCAGTGAGTTAAGGATCCAGTG |
| **RPL32** | R: CGAGCCCACTATTCATTTCAAC |
|  | Probe: 56-FAM/TGTGGCAGA/ZEN/TGTGGTTTAGACCCC/IBFQ |
