# Supplementary material for: Salmonella enterica Serovar Typhimurium Exploits Inflammation to Modify Swine Intestinal Microbiota
Source: Front Cell Infect Microbiol. 2016 Jan 22;5:106. doi: 10.3389/fcimb.2015.00106 (PMC4722131; doi:10.3389/fcimb.2015.00106)
Supplement: Supplementary file 2 [file Table2.docx]

**Supplementary Table 2.** Real-time PCR primers used in this study. The primer sets were tested for sensitivity and specificity against a panel of genomic DNAs and showed minimal or no cross-reactivity.

| Group | Primer | Sequence (5’ to 3’) | References |
| --- | --- | --- | --- |
| Eubacteria | UniF340  UniR514 | ACTCCTACGGGAGGCAGCAGT  ATTACCGCGGCTGCTGGC | M. Barman D. Unold, K. Shifley, E. Amir, K. Hung, N. Bos, and N. Salzman et al. Infect Immun. **76**:907–15, 2008. |
| Lactobacillus/Lactococcus | LabF362  LabR677 | AGCAGTAGGGAATCTTCCA  CACCGCTACACATGGAG |  |
| Eubacterium rectale/ Clostridium coccoides | UniF338  C.cocR491 | ACTCCTACGGGAGGCAGC  GCTTCTTAGTCAGGTACCGTCAT |  |
| Bacteroides | BactF285  UniR338 | GGTTCTGAGAGGAGGTCCC  GCTGCCTCCCGTAGGAGT |  |
| Enterobacteriaceae | UniF515  Ent826R | GTGCCAGCMGCCGCGGTAA  GCCTCAAGGGCACAACCTCCAAG |  |
| Salmonella | Sal454  Uni785R | TGTTGTGGTTAATAACCGCA  GACTACCAGGGTATCTAATCC |  |
| Bifidobacteria | F-Bifid 09c  R-Bifid 06 | CGGGTGAGTAATGCGTGACC  TGATAGGACGCGACCCCA | J. P. Furet, O. [Firmesse](http://www.ncbi.nlm.nih.gov/pubmed/?term=Firmesse%20O%5BAuthor%5D&cauthor=true&cauthor_uid=19302550), M. [Gourmelon](http://www.ncbi.nlm.nih.gov/pubmed/?term=Gourmelon%20M%5BAuthor%5D&cauthor=true&cauthor_uid=19302550), C. [Bridonneau](http://www.ncbi.nlm.nih.gov/pubmed/?term=Bridonneau%20C%5BAuthor%5D&cauthor=true&cauthor_uid=19302550), J. [Tap](http://www.ncbi.nlm.nih.gov/pubmed/?term=Tap%20J%5BAuthor%5D&cauthor=true&cauthor_uid=19302550), S. [Mondot](http://www.ncbi.nlm.nih.gov/pubmed/?term=Mondot%20S%5BAuthor%5D&cauthor=true&cauthor_uid=19302550), J. [Doré](http://www.ncbi.nlm.nih.gov/pubmed/?term=Dor%C3%A9%20J%5BAuthor%5D&cauthor=true&cauthor_uid=19302550), and G. [Corthier](http://www.ncbi.nlm.nih.gov/pubmed/?term=Corthier%20G%5BAuthor%5D&cauthor=true&cauthor_uid=19302550), [FEMS Microbiol Ecol.](http://www.ncbi.nlm.nih.gov/pubmed/19302550) **68**:351-62, 2009. |
| Prevotella | PrevF  PrevR | CGGGTTGTAAACTGCTTTTATGAAG  TTTATTGGGTTTAAAGGGAGCG | S. M. D. Bearson, H. K. Allen, B. L. Bearson, T. Looft, B. W. Brunelle et al, Infect Genet Evol. **16**:330-40, 2013. |
