# Supplementary material for: Salmonella enterica Serovar Typhimurium Exploits Inflammation to Modify Swine Intestinal Microbiota
Source: Front Cell Infect Microbiol. 2016 Jan 22;5:106. doi: 10.3389/fcimb.2015.00106 (PMC4722131; doi:10.3389/fcimb.2015.00106)
Supplement: Supplementary file 5 [file DataSheet1.docx]

***Salmonella* *enterica* serovar Typhimurium exploits inflammation to modify swine intestinal microbiota.**

Rosanna Drumo, Michele Pesciaroli, Jessica Ruggeri, Michela Tarantino, Barbara Chirullo, Claudia Pistoia, Paola Petrucci, Nicola Martinelli, Livia Moscati, Elisabetta Manuali, Silvia Pavone, Matteo Picciolini, Serena Ammendola, Gianfranco Gabai, Andrea Battistoni, Giovanni Pezzotti, Giovanni Loris Alborali, Valerio Napolioni, Paolo Pasquali^*^, Chiara F. Magistrali^*^.

Correspondence: Paolo Pasquali: [paolo.pasquali@iss.it](mailto:paolo.pasquali@iss.it)

Chiara Francesca Magistrali: [c.magistrali@izsum.it](mailto:c.magistrali@izsum.it)

**Supplementary Figures**





**Supplementary Figure 1.** **STM^wt^ induces a higher colonization than STM^ΔznuABC^ at 12 dpi.** Piglets were orally infected with 2×10^9^ CFU of STM^ΔznuABC^ (group B) or STM^wt^ (group C) and bacterial burdens were determined at 12 dpi. Differences between groups B and C were considered significant when *P*≤0.05 (*), Mann Whitney test. Error bars indicate one SD from the mean.





**Supplementary Figure 2 (A-H).** TNF-α, IL1-α, IL1-β and INF-γ expression was measured in the colon at 1 and 12 dpi, by real time RT-PCR. Grey bars and black bars represent STM^ΔznuABC^- (group B) and STM^wt^-infected piglets (group C), respectively. The asterisks indicate statistical significance **P*≤0.05 and ***P*≤0.01), Mann-Whitney test.





**Supplementary Figure 3 (A-B).** TNF-α, IL1-α, IL1-β and INF-γ expression in the ileocecal lymph nodes at 1 and 12 dpi, was measured by real time RT-PCR. Grey bars and black bars represent, STM^ΔznuABC^ -(group B) and STM^wt^ -infected piglets (group C), respectively. The asterisk indicates statistical significance **P*≤0.05, Mann-Whitney test.





**Supplementary Figure 4 (A-E).** Quantitative analysis of specific commensal bacterial groups in the feces of piglets infected with STM^ΔznuABC^ or STM^wt^ at different timepoints. White bars represent uninfected controls. Grey bars and grey-black bars represent STM^ΔznuABC^- (group B) and STM^wt^-infected piglets (group C), respectively. P-values were calculated using one-way ANOVA with Bonferroni’s post-test. Significant differences between groups are indicated by **P*≤0.05, ***P*≤0.01 and ****P*≤0.001. Eub, all bacteria; Lacto, *Lactobacillus/Lactococcus* group; Clost, *Eubacterium rectale/Clostridium coccoides*; Bact, *Bacteroides* sp.; Bifido, *Bifidobacterium*; Prev, *Prevotellaceae*; Ent, *Enterobacteriaceae* other than *Salmonella*; STM, *S. enterica* serovar Typhimurium.





**Supplementary Figure 5 (A-B).** Quantitative analysis of specific commensal bacterial groups in the colonic content of piglets infected with STM^ΔznuABC^ or STM^wt^, at 1 and 12 dpi. White bars represent uninfected controls. Grey bars and grey-black bars represent STM^ΔznuABC^- (group B) and STM^wt^-infected piglets (group C), respectively. P-values were calculated using one-way ANOVA with Bonferroni’s post-test. Significant differences between groups are indicated by **P*≤0.05), ***P*≤0.01) and ****P*≤0.001. Eub, all bacteria; Lacto, *Lactobacillus/Lactococcus* group; Clost, *Eubacterium rectale/Clostridium coccoides*; Bact, *Bacteroides* sp.; Bifido, *Bifidobacterium*; Prev, *Prevotellaceae*; Ent, *Enterobacteriaceae* other than *Salmonella*; STM, *S. enterica* serovar Typhimurium.


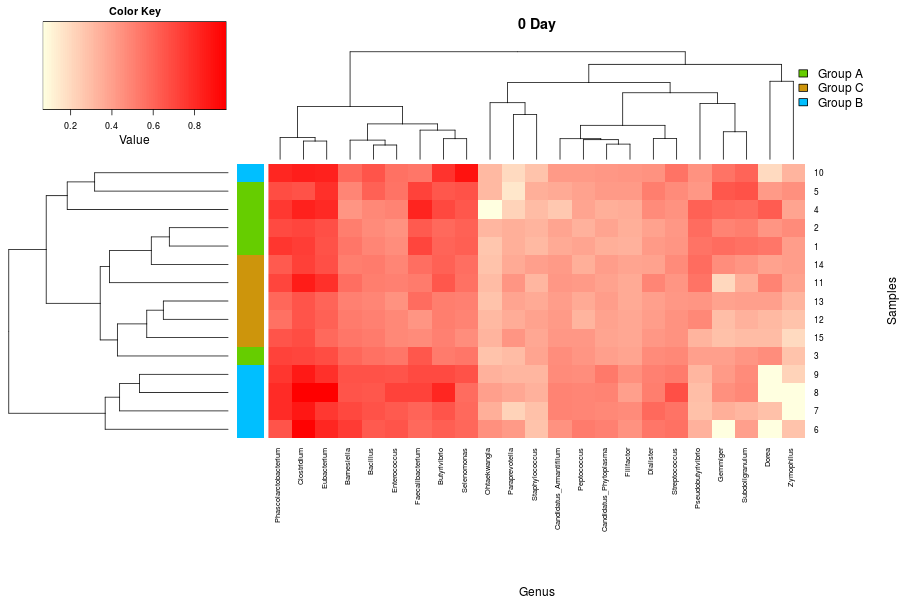


**Supplementary Figure 6.** Heatmap indicating genus-level changes in the microbiota composition of piglets naive (group A), and piglets infected with STM^ΔznuABC^ (group B) or with STM^wt^ (group C) at 2 and 12 dpi. The relative abundance of the most represented genera is indicated by a gradient of color from white (low abundance) to red (high abundance). The hierarchical clustering analysis of the samples, based on the similarity of the microbiota composition, are displayed on the left. Animals 1-5: group A (Naïve), green; animals 6 -10: group B (STM^ΔznuABC^), blue; piglets 11-15: group C (STM^wt^), orange.

**Supplementary Tables.**

**Supplementary Table 3.** Genus-level normalized data.

**Supplementary Table 4.** Relative abundances of taxa that resulted significantly different across the three groups at 2 dpi and 12 dpi.
